# Freshman College Students’ Reasons for Enrolling in and Anticipated Benefits from a Basic College Physical Education Activity Course

**DOI:** 10.3389/fpubh.2015.00162

**Published:** 2015-06-24

**Authors:** Jeremy Lackman, Matthew Lee Smith, Elisa Beth McNeill

**Affiliations:** ^1^EARTH University, Limon, Costa Rica; ^2^Department of Health Promotion and Behavior, College of Public Health, The University of Georgia, Athens, GA, USA; ^3^Department of Health and Kinesiology, Texas A&M University, College Station, TX, USA

**Keywords:** college student, physical activity, fitness, course enrollment

## Abstract

**Background:**

Given the rise in US obesity rates in adulthood, efforts are needed to assess physical activity engagement during the college years as a strategy to promote a lifetime of being physically active. This study identifies the reasons incoming college freshman enrolled in basic physical education activity courses (BPEAC) and the perceived benefits they anticipated receiving as a result of course participation.

**Methods:**

Data collected from 302 college freshmen in September 2013 were analyzed. A paper-based questionnaire was administered to 78% of BPEAC sections offered at a large Southeastern University. Frequencies were presented for all participants, which were then compared by sex and course type. Kappa statistics were calculated to examine the concordance between participants’ reasons for enrolling in the course and the benefits they anticipated from course enrollment.

**Results:**

Diverse physical, mental, social, and academic reasons for enrolling in BPEAC were reported by study participants. Varied anticipated benefits from course participation were reported as well. Reported enrollment reasons and anticipated benefits differed by sex and course type. High concordance between matched enrollment reasons and anticipated benefits was observed.

**Conclusion:**

Implications highlight the need for universities to provide quality BPEAC, promote high-quality instruction, and offer a wide variety of physical education courses to meet the diverse needs of students.

## Introduction

Physical activity is an important component of lifetime health and wellness. Substantial research links the effects of physical activity to positive physiological and psychological health outcomes (1, 2). Physical activity is essential for obesity prevention (3), and regular exercise is linked to reduced risk of chronic diseases, such as cardiovascular disease, Type-II diabetes, osteoporosis, and cancer (4–6). Physical activity has also been identified as an effective antidepressant and a promising treatment for depression among healthy individuals (7, 8).

While physical activity is essential during all life stages, engagement in physical activity decreases with age (9). The most significant exercise declines occur during late adolescence and young adulthood (10–13), and the sharpest physical activity declines occur immediately following high school (14). Approximately 50% of all college students report decreased participation in physical activity following high-school graduation (15), and physical activity patterns among college students are insufficient to improve health and fitness (15).

To maximize the health and fitness benefits of physical activity, it is recommended that individuals engage in moderate to vigorous activity most days a week (16). Weekly, adults should participate in 150 min of moderate-intensity aerobic activity, such as walking briskly, with two or more days a week dedicated to muscle-strengthening activities like weight lifting (17). Many young adults on college campuses are not meeting physical activity recommendations and substantial proportions are leading sedentary lifestyles (18). College students spend considerable time each day sitting and/or being sedentary, which is essentially preparing them for sedentary careers following graduation (19). Greater attention should be paid to students’ health and physical activity habits to help them establish positive lifetime health and physical activity trajectories (20). College campuses have the unique opportunity for targeted promotional strategies to encourage student participation in physical activity classes, laying the foundation for continued participation after college graduation.

Venues to advance awareness and promote healthy behavior choices are essential in the efforts to help students achieve positive health outcomes. University settings are an ideal setting for promoting lifestyle change among a captive audience (21). Colleges afford students many opportunities to engage in physical activity, such as intramural sports, activity classes, campus recreation, and club or varsity sports (22). Many universities offer basic activity courses as electives while others require enrollment in a minimum number of such credit hours for graduation. Studies have shown enrolling in an activity class can impact attitudes and knowledge about health risk behaviors and lifestyle factors necessary for chronic disease prevention (23, 24). In addition to the obvious benefits of activity course enrollment, physical health, and academic requirements, these courses can improve students’ mental health and self-esteem, as well as provide an opportunity for social interaction with peers. Evidence suggests college alumni who participated in activity courses had better lifestyle habits compared to students who did not enroll in such courses (24, 25).

College students enroll in physical activity classes for a variety of reasons. Reported reasons include to have fun/enjoyment, learn new skills, work out/exercise regularly, earn credit, be social, challenge themselves, and improve their previous skills (among other reasons) (26–34). Reasons for enrolling in physical activity courses may differ between males and females and based on the type or nature of the class (fitness-oriented versus sport-oriented). For example, males have been found to participate more in team sports compared to females who are more likely to enroll in classes aimed at enhancing fitness (26, 27, 34–36). While these studies offer basic insight into reasons for course enrollment, a greater understanding is needed about the health-related drivers associated with physical activity course selection. Little is known about why incoming freshman college students enroll in basic physical education courses or what they expect to benefit from enrollment. Therefore, the purposes of this study were to: (1) identify the reasons incoming college freshman enrolled in basic physical education activity courses; (2) identify the perceived benefits they anticipated receiving as a result of participating in the course; and (3) assess the concordance between reasons for enrolling and anticipated benefits.

## Materials and Methods

### Sport and fitness courses

This study was conducted at a large Southeastern University that offers two types of basic physical education activity courses (BPEAC): sport and fitness. These sport and fitness courses provide students an opportunity to engage in physical activity, learn new skills and activities, and develop an appreciation for healthy lifestyles (27). Sport courses primarily emphasize skill development and game play, which are offered at the beginning or intermediate level. Examples of sport courses include basketball, bowling, soccer, tennis, and volleyball. Fitness courses are designed to enhance fitness levels, convey knowledge, and promote life-long physical activity and wellness. Examples of fitness courses include outdoor adventure, self-defense, aerobic dance, walking, and weight management. Approximately 100 sections of BPEAC are offered each Fall and Spring semester.

All BPEAC can be offered either indoors or outdoors. Courses convene either twice per week for 1.25 h or three times per week for 50 min. BPEAC are graded on a pass/fail basis and taught by teaching assistants or instructors employed by the university. Students are evaluated based on their completion of required readings, quizzes, attendance, and goal setting activities. To be eligible for graduation at this Southeastern University, all undergraduate students are required to pass a one credit hour BPEAC.

### Participants and procedures

Participants were freshman recruited from 77 of the 99 (77.8%) BPEAC sections offered at the university during the Fall 2013 semester. Researchers emailed all BPEAC teaching assistants and instructors to request permission to collect data during their class. Informed consent was obtained and instruments were administered. No incentives were provided to students for participation. Institutional Review Board approval was received for this study.

Of the 77 BPEAC sections visited, 39 were sport and 38 were fitness. A total of 389 freshmen were in attendance on the days data were collected. Four students declined to participate. Of the 385 students who returned instruments, 1 was omitted because over 50% of the requested data were not provided. Additional cases were further omitted from the available sample of 384 freshmen. More specifically, 5 cases were omitted because students reported being in a class not classified as sport or fitness (i.e., self-defense), 1 case was omitted for missing data about the participants gender, 16 cases were omitted for missing data about family income, and the remaining 60 cases were omitted for missing data on one or more of the 30 reason- or benefit-related items. The final analytic sample consisted of 302 college freshmen. We compared study participants to freshman enrollment data available on the university’s website, and determined that participants were representative of the freshman class in terms of sex and race/ethnicity.

### Instrument

Using a modified version of a previously validated instrument (26, 27, 35), data were collected about students’ perceptions concerning enrollment and anticipated benefits related to physical education activity courses. The instrument contained close-ended items using Likert-type scales and lists asking participants to check all responses that apply. Instruments were distributed during class time and took approximately 10 min for each participant to complete.

### Measures

#### Demographic Characteristics

To identify demographic characteristics of student participants, socio-demographic variables in this study included age, sex, and race/ethnicity. Participants were also asked to report their family’s household income using predefined categories and the rural designation of their high school (i.e., rural, suburban, urban).

#### Physical Activity Engagement and Perceptions

Respondents were asked to report information about their past and current engagement in physical activity and perceptions about physical activity. To assess past physical activity engagement, participants were asked to report the number of physical education classes they completed in high school. To assess current physical activity engagement, participants were queried to report if they participate in intramural sports and if they engage in physical activity outside of class time. To assess perceptions about physical activity, participants reported how important they believed physical activity to be (four-point Likert-type scale ranging from “not important” to “very important”). Participants were also asked to compare their level of physical activity to others of the same age and sex (five-point Likert-type scale ranging from “much less active” to “much more active”). Finally, participants were asked if they believed participation in the BPEAC would influence their future physical activity and if they plan to enroll in another BPEAC in the future.

#### Reasons for BPEAC Enrollment

Using a “check all that apply” list of 21 items, participants were asked to indicate reasons why they enrolled in the BPEAC. Each of the 21 reasons was recorded independently as “endorsed” or “not endorsed.” For the purposes of this study, reasons for enrollment were organized in categories including general reasons (*n* = 6 items), physical reasons (*n* = 6 items), mental reasons (*n* = 4 items), social reasons (*n* = 2 items), and academic reasons (*n* = 3 items). All endorsed variables were summed for each participant to create a continuous variable indicating the number of reasons they enrolled in the course.

#### Benefits of BPEAC Enrollment

Using a “check all that apply” list of 19 items, participants were asked to indicate the benefits they anticipated receiving because of enrollment in the BPEAC. Each of the 19 reasons was recorded independently as “endorsed” or “not endorsed.” For the purposes of this study, reasons for enrollment were organized in categories including general reasons (*n* = 7 items), physical reasons (*n* = 4 items), mental reasons (*n* = 3 items), social reasons (*n* = 3 items), and academic reasons (*n* = 2 items). All endorsed variables were summed for each participant to create a continuous variable indicating the number of benefits anticipated from enrolling in the course.

### Data analyses

Statistical analyses for this study were performed using SPSS (version 21). Based on variation in course objectives, participant responses were compared by participants’ sex and course type. As such, frequencies were calculated for all study variables, which were initially examined in relationship to respondents’ sex (i.e., male, female) and the type of BPEAC in which they were enrolled (i.e., sport, fitness). Pearson’s chi-square tests were performed to assess the independence between these categories and categorized study variables. Independent sample *t*-tests were used to examine mean differences for continuous variables based on sex and course type. To the extent possible, concepts related to reasons for enrolling in BPEAC and anticipated benefits were matched. Concordance for matching concepts between reasons and benefits was evaluated using kappa statistics. A total of 11 matched variables were assessed using categories including physical reasons (*n* = 3 items), mental reasons (*n* = 4 items), social reasons (*n* = 1 items), and academic reasons (*n* = 3 items).

## Results

### Sample characteristics

Sample characteristics of the 302 study participants are presented in Table 1. Of these participants, 68.2% were female and 65.6% were enrolled in a sport BPEAC. On average, participants were aged 18.07 (±0.49) years. The majority of participants was non-Hispanic white (76.5%) and attended high school in a suburban area (68.9%). On average, participants reported taking 1.98 (±1.51) physical education classes in high school. Most participants reported physical activity as being important (37.1%) or very important (52.6%). Approximately 17% of participants reported their physical activity level was less than others their same age and sex, 30.8% reported their activity level was approximately the same as others their same age and sex, and 52.7% reported their physical activity level was more than others their same age and sex. Engagement in physical activity outside of class was reported by 87% of participants, and 24.8% reported participating in intramural sports. When asked about future BPEAC enrollment, 44.7% reported they plan to take another BPEAC. About 60% of participants reported that participation in the BPEAC will somewhat influence their future physical activity and 24.8% reported it would influence their future physical activity a lot.

**Table 1:** **Sample characteristics and physical activity engagement among college students enrolled in asic physical education and activity courses**.

		Sex				Course type			
	Total (*n* = 302)	Male (*n* = 96)	Female (*n* = 206)	χ^2^ or *t*	*P*	Sport (*n* = 198)	Fitness (*n* = 104)	χ^2^ or *t*	*P*
**Sex**								17.44	<0.001
Male	31.8%	–	–	–	–	39.9%	16.3%		
Female	68.2%	–	–			60.1%	83.7%		
**Age**	18.07 (±0.49)	18.02 (±0.36)	18.01 (±0.36)	0.25	0.800	18.00 (±0.35)	18.06 (±0.36)	−1.58	0.115
**Race/ethnicity**				1.54	0.820			4.77	0.312
Other	1.0%	0.0%	1.5%			1.5%	0.0%		
Hispanic or Latino	2.6%	3.1%	2.4%			3.0%	1.9%		
Asian or Pacific Islander	10.3%	10.4%	10.2%			12.1%	6.7%		
Black or African-American	9.6%	9.4%	9.7%			10.1%	8.7%		
Non-Hispanic White	76.5%	77.1%	76.2%			73.2%	82.7%		
**High school rurality**				3.68	0.159			1.05	0.593
Rural	15.9%	13.5%	17.0%			16.2%	15.4%		
Suburban	68.9%	76.0%	65.5%			67.2%	72.1%		
Urban	15.2%	10.4%	17.5%			16.7%	12.5%		
**Family’s Household Income**				2.21	0.530			2.87	0.413
<$50,000	17.9%	15.6%	18.9%			16.2%	21.2%		
$50,000–$69,999	14.6%	11.5%	16.0%			15.2%	13.5%		
$70,000–$89,999	19.9%	19.8%	19.9%			18.2%	23.1%		
$90,000 or more	47.7%	53.1%	45.1%			50.5%	42.3%		
**Number of physical education classes in high school**	1.98 (±1.51)	2.56 (±1.73)	1.68 (±1.33)	4.40	<0.001	2.02 (±1.55)	1.86 (±1.46)	−1.58	0.115
**Importance of physical activity participation**				0.62	0.892			0.66	0.882
Not important	0.7%	1.0%	0.5%			0.5%	1.0%		
Somewhat important	9.6%	8.3%	10.2%			9.1%	10.6%		
Important	37.1%	36.5%	37.4%			36.4%	38.5%		
Very important	52.6%	54.2%	51.9%			54.0%	50.0%		
**Engage in physical activity outside class**				0.60	0.438			1.13	0.287
No	12.6%	10.4%	13.6%			11.1%	15.4%		
Yes	87.4%	89.6%	86.4%			88.9%	84.6%		
**Participate in intramural sports**				26.98	<0.001			15.02	<0.001
No	75.2%	56.3%	84.0%			68.2%	88.5%		
Yes	24.8%	43.8%	16.0%			31.8%	11.5%		
**Physical activity compared to others (same age/sex)**				17.91	0.001			5.44	0.245
Much less active	3.0%	0.0%	4.4%			1.5%	5.8%		
Somewhat less active	13.6%	10.4%	15.0%			13.1%	14.4%		
About the same	30.8%	24.0%	34.0%			30.3%	31.7%		
Somewhat more active	42.4%	46.9%	40.3%			43.4%	40.4%		
Much more active	10.3%	18.8%	6.3%			11.6%	7.7%		
**Participation in PEDB course will influence future physical activity**				11.32	0.003			1.42	0.493
Not at all	14.9%	25.0%	10.2%			16.7%	11.5%		
Somewhat	60.3%	53.1%	63.6%			59.1%	62.5%		
A lot	24.8%	21.9%	26.2%			24.2%	26.0%		
**Plan to take another PEDB course**				1.60	0.206			0.73	0.393
No	55.3%	50.0%	57.8%			57.1%	51.9%		
Yes	44.7%	50.0%	42.2%			42.9%	48.1%		

When comparing study variables by the participant’s sex, a significantly larger proportion of male students were enrolled in sport BPEAC relative to a larger proportion of females enrolled in fitness BPEAC (χ^2^ = 17.44, *P* < 0.001). On average, males reported taking more physical activity classes in high school (*t* = 4.40, *P* < 0.001). Relative to females, a significantly larger proportion of males reported being more physically active than others their same age and sex (χ^2^ = 17.91, *P* = 0.001). A larger proportion of males reported participating in intramural sports (χ^2^ = 26.98, *P* < 0.001). However, a significantly larger proportion of females reported participation in the BPEAC would influence their future physical activity (χ^2^ = 11.32, *P* = 0.003). When comparing study variables by the participant’s course type, a significantly larger proportion of participants enrolled in a sport BPEAC reported participating in intramural sports (χ^2^ = 15.02, *P* < 0.001).

Reported reasons for enrolling in BPEAC are presented by category in Table 2. In terms of general reasons for enrollment, the most prevalent reasons endorsed were to “have fun” (64.2%), “have structured exercise time” (47.0%), and “learn a new activity” (32.5%). In terms of physical reasons, the most prevalent reasons endorsed were to “exercise regularly” (67.2%) and “improve my fitness level” (65.2%). For mental reasons, the most endorsed reasons for enrollment were to “reduce stress/anxiety levels” (35.1%) and “relax” (28.5%). The most endorsed social reason for enrollment was to “meet new people” (45.7%). In terms of academic reasons, the most prevalent reasons endorsed were to “earn credit” (87.4%) and fulfill a graduation requirement (75.8%). On average, 7.44 (±3.26) reasons were reported for enrolling in BPEAC courses.

**Table 2:** **Reasons why college freshmen enrolled in basic physical education and activity courses**.

		Sex				Course type			
	Total (*n* = 302)	Male (*n* = 96)	Female (*n* = 206)	χ^2^ or *t*	*P*	Sport (*n* = 198)	Fitness (*n* = 104)	χ^2^ or *t*	*P*
**GENERAL REASONS**									
**To learn about the health benefits of physical activity**				1.88	0.171			7.89	0.005
Not endorse	91.7%	88.5%	93.2%			94.9%	85.6%		
Endorse	8.3%	11.5%	6.8%			5.1%	14.4%		
**To learn a new activity**				5.84	0.016			23.52	<0.001
Not endorse	67.5%	77.1%	63.1%			58.1%	85.6%		
Endorse	32.5%	22.9%	36.9%			41.9%	14.4%		
**To learn physical activity habits for the future**				3.30	0.069			5.92	0.015
Not endorse	86.4%	91.7%	84.0%			89.9%	79.8%		
Endorse	13.6%	8.3%	16.0%			10.1%	20.2%		
**To have structured exercise time**				12.26	<0.001			7.25	0.007
Not endorse	53.0%	67.7%	46.1%			58.6%	42.3%		
Endorse	47.0%	32.3%	53.9%			41.4%	57.7%		
**To participate in a competitive activity**				7.45	0.006			34.46	<0.001
Not endorse	79.1%	69.8%	83.5%			69.2%	98.1%		
Endorse	20.9%	30.2%	16.5%			30.8%	1.9%		
**To have fun**				1.25	0.264			49.37	<0.001
Not endorse	35.8%	31.3%	37.9%			21.7%	62.5%		
Endorse	64.2%	68.8%	62.1%			78.3%	37.5%		
**PHYSICAL REASONS**									
**To exercise regularly**				23.80	<0.001			13.22	<0.001
Not endorse	32.8%	52.1%	23.8%			39.9%	19.2%		
Endorse	67.2%	47.9%	76.2%			60.1%	80.8%		
**To develop sport skills**				0.16	0.687			60.27	<0.001
Not endorse	67.2%	65.6%	68.0%			52.0%	96.2%		
Endorse	32.8%	34.4%	32.0%			48.0%	3.8%		
**To develop fitness skills**				4.51	0.034			1.25	0.264
Not endorse	76.8%	84.4%	73.3%			78.8%	73.1%		
Endorse	23.2%	15.6%	26.7%			21.2%	26.9%		
**To maintain my body weight**				13.73	<0.001			6.34	0.012
Not endorse	68.9%	83.3%	62.1%			73.7%	59.6%		
Endorse	31.1%	16.7%	37.9%			26.3%	40.4%		
**To lose weight**				12.29	<0.001			20.45	<0.001
Not endorse	79.8%	91.7%	74.3%			87.4%	65.4%		
Endorse	20.2%	8.3%	25.7%			12.6%	34.6%		
**To improve my fitness level (“get in shape”) or (“stay in shape”)**				10.73	0.001			9.56	0.002
Not endorse	34.8%	47.9%	28.6%			40.9%	23.1%		
Endorse	65.2%	52.1%	71.4%			59.1%	76.9%		
**MENTAL REASONS**									
**To relax**				3.33	0.068			0.37	0.545
Not endorse	71.5%	64.6%	74.8%			69.2%	76.0%		
Endorse	28.5%	35.4%	25.2%			30.8%	24.0%		
**To reduce stress/anxiety levels**				6.30	0.012			0.02	0.900
Not endorse	64.9%	75.0%	60.2%			65.2%	64.4%		
Endorse	35.1%	25.0%	39.8%			34.8%	35.6%		
**To improve my self-confidence**				0.04	0.848			0.02	0.893
Not endorse	90.1%	89.6%	90.3%			89.9%	90.4%		
Endorse	9.9%	10.4%	9.7%			10.1%	9.6%		
**To enhance my self-image**				3.92	0.048			20.43	<0.001
Not endorse	88.4%	93.8%	85.9%			94.4%	76.9%		
Endorse	11.6%	6.3%	14.1%			5.6%	23.1%		
**SOCIAL REASONS**									
**To participate with a friend**				0.99	0.320			2.87	0.090
Not endorse	89.1%	86.5%	90.3%			86.9%	93.3%		
Endorse	10.9%	13.5%	9.7%			13.1%	6.7%		
**To meet new people**				0.92	0.337			20.28	<0.001
Not endorse	54.3%	58.3%	52.4%			44.9%	72.1%		
Endorse	45.7%	41.7%	47.6%			55.1%	27.9%		
**ACADEMIC REASONS**									
**To earn credit**				6.65	0.010			0.11	0.739
Not endorse	12.6%	19.8%	9.2%			12.1%	13.5%		
Endorse	87.4%	80.2%	90.8%			87.9%	86.5%		
**Because it is included in my tuition**				0.40	0.526			0.89	0.345
Not endorse	90.1%	91.7%	89.3%			88.9%	92.3%		
Endorse	9.9%	8.3%	10.7%			11.1%	7.7%		
**Because it is required to graduate**				2.80	0.094			0.37	0.545
Not endorse	24.2%	30.2%	21.4%			25.3%	22.1%		
Endorse	75.8%	69.8%	78.6%			74.7%	77.9%		
**Number of reasons enrolled in course**	7.44 (±3.26)	6.40 (±3.10)	7.88 (±3.23)	−3.77	<0.001	7.58 (±3.40)	7.09 (±2.97)	1.253	0.211

When comparing general reasons for BPEAC enrollment by sex, a significantly larger proportion of females reported to “learn a new activity” (χ^2^ = 5.84, *P* = 0.016) and “have structured exercise time” (χ^2^ = 12.26, *P* < 0.001) as reasons for enrollment. Conversely, a significantly larger proportion of males reported to “participate in a competitive activity” as a reason for enrollment (χ^2^ = 7.45, *P* = 0.006). When comparing physical reasons for BPEAC enrollment by sex, a significantly larger proportion of females endorsed five of the six physical reasons. For mental reasons, a significantly larger proportion of females reported to “reduce stress/anxiety levels” (χ^2^ = 6.30, *P* = 0.012) and “enhance my self-image” (χ^2^ = 3.92, *P* = 0.048) as reasons for enrolling in BPEAC. When comparing academic reasons for BPEAC enrollment by sex, a significantly larger proportion of females reported to “earn credit” (χ^2^ = 6.65, *P* = 0.010) as a reason for enrollment. Overall, on average, females endorsed significantly more reasons for enrolling in BPEAC (*t* = −3.77, *P* < 0.001).

When comparing general reasons for BPEAC enrollment by course type, a significantly larger proportion of those in fitness courses reported to “learn about the health benefits of physical activity” (χ^2^ = 7.89, *P* = 0.005), “learn physical activity habits for the future” (χ^2^ = 5.92, *P* = 0.015), and “have structured exercise time” (χ^2^ = 7.25, *P* = 0.007) as reasons for enrollment. Conversely, a significantly larger proportion of those in sport classes reported to “learn a new activity” (χ^2^ = 23.52, *P* < 0.001), “participate in a competitive activity” (χ^2^ = 34.46, *P* < 0.001), and “have fun” (χ^2^ = 49.37, *P* < 0.001) as reasons for enrollment. When comparing physical reasons for BPEAC enrollment by course type, a significantly larger proportion of those in fitness classes endorsed to “exercise regularly” (χ^2^ = 13.22, *P* < 0.001), “maintain my body weight” (χ^2^ = 6.34, *P* = 0.012), “lose weight” (χ^2^ = 20.45, *P* < 0.001), and “improve my fitness level” (χ^2^ = 9.56, *P* = 0.002) as reasons for enrollment. Conversely, a significantly larger proportion of those in sport courses reported to “develop sport skills” as a reason for enrollment (χ^2^ = 60.27, *P* < 0.001). For mental reasons, a significantly larger proportion of those in fitness courses reported to “enhance my self-image” (χ^2^ = 20.43, *P* < 0.001) as a reason for enrolling in BPEAC. When comparing social reasons for BPEAC enrollment by course type, a significantly larger proportion of those in sport courses reported to “meet new people” (χ^2^ = 20.28, *P* < 0.001) as a reason for enrollment.

Anticipated benefits from enrolling in BPEAC are presented by category in Table 3. In terms of general benefits for enrollment, the most prevalent benefits endorsed were to “allow me to have fun” (63.6%), “keep me healthy” (58.6%), and “improve my health” (57.9%). The most endorsed anticipated physical benefit was to “help me stay active” (84.1%). For mental benefits, the most endorsed benefits were to “reduce stress” (46.0%) and “help me relax” (32.8%). The most endorsed anticipated social benefits from enrollment were to “make new friends” (54.3%) and “allow me to meet new people” (49.0%). In terms of academic benefits, the most prevalent benefit endorsed was to “allow me to earn credit” (83.8%). On average, 9.01 (±4.06) anticipated benefits were reported from enrolling in BPEAC.

**Table 3:** **College Freshmen’s anticipated benefits from enrolling in basic physical education and activity courses**.

		Sex				Course type			
	Total (*n* = 302)	Male (*n* = 96)	Female (*n* = 206)	χ^2^ or *t*	*P*	Sport (*n* = 198)	Fitness (*n* = 104)	χ^2^ or *t*	*P*
**GENERAL BENEFITS**									
**Help me feel better**				2.11	0.146			3.54	0.060
Not endorse	63.9%	69.8%	61.2%			67.7%	56.7%		
Endorse	36.1%	30.2%	38.8%			32.3%	43.3%		
**Keep me healthy**				18.76	<0.001			13.69	<0.001
Not endorse	41.4%	59.4%	33.0%			49.0%	26.9%		
Endorse	58.6%	40.6%	67.0%			51.0%	73.1%		
**Improve my health**				7.08	0.008			23.44	<0.001
Not endorse	42.1%	53.1%	36.9%			52.0%	23.1%		
Endorse	57.9%	46.9%	63.1%			48.0%	76.9%		
**Learn new physical activity skills**				0.03	0.863			31.99	<0.001
Not endorse	57.6%	58.3%	57.3%			46.0%	79.8%		
Endorse	42.4%	41.7%	42.7%			54.0%	20.2%		
**Learn about sports**				0.42	0.518			51.08	<0.001
Not endorse	70.2%	67.7%	71.4%			56.6%	96.2%		
Endorse	29.8%	32.3%	28.6%			43.4%	3.8%		
**Provide me with a challenge**				0.01	0.946			13.66	<0.001
Not endorse	65.9%	65.6%	66.0%			58.6%	79.8%		
Endorse	34.1%	34.4%	34.0%			41.4%	20.2%		
**Allow me to have fun**				0.58	0.446			39.96	<0.001
Not endorse	36.4%	33.3%	37.9%			23.7%	60.6%		
Endorse	63.6%	66.7%	62.1%			76.3%	39.4%		
**PHYSICAL BENEFITS**									
**Help me stay active**				10.84	0.001			2.25	0.134
Not endorse	15.9%	26.0%	11.2%			18.2%	11.5%		
Endorse	84.1%	74.0%	88.8%			81.8%	88.5%		
**Help control my weight**				23.35	<0.001			25.27	<0.001
Not endorse	62.6%	82.3%	53.4%			72.7%	43.3%		
Endorse	37.4%	17.7%	46.6%			27.3%	56.7%		
**Improve my aerobic fitness level**				12.94	<0.001			9.05	0.003
Not endorse	57.9%	72.9%	51.0%			64.1%	46.2%		
Endorse	42.1%	27.1%	49.0%			35.9%	53.8%		
**Improve my strength**				3.54	0.060			0.00	0.977
Not endorse	73.2%	80.2%	69.9%			73.2%	73.1%		
Endorse	26.8%	19.8%	30.1%			26.8%	26.9%		
**MENTAL BENEFITS**									
**Help me relax**				3.93	0.047			0.08	0.778
Not endorse	67.2%	59.4%	70.9%			66.7%	68.3%		
Endorse	32.8%	40.6%	29.1%			33.3%	31.7%		
**Reduce my stress**				1.08	0.299			0.49	0.486
Not endorse	54.0%	58.3%	51.9%			52.5%	56.7%		
Endorse	46.0%	41.7%	48.1%			47.5%	43.3%		
**Improve my self-confidence**				1.73	0.189			4.39	0.036
Not endorse	88.1%	91.7%	86.4%			90.9%	82.7%		
Endorse	11.9%	8.3%	13.6%			9.1%	17.3%		
**SOCIAL BENEFITS**									
**Provide me with a social outlet**				0.43	0.514			5.61	0.018
Not endorse	69.2%	66.7%	70.4%			64.6%	77.9%		
Endorse	30.8%	33.3%	29.6%			35.4%	22.1%		
**Allow me to meet new people**				1.56	0.212			4.72	0.030
Not endorse	51.0%	56.3%	48.5%			46.5%	59.6%		
Endorse	49.0%	43.8%	51.5%			53.5%	40.4%		
**Make new friends**				0.08	0.779			14.16	<0.001
Not endorse	45.7%	46.9%	45.1%			37.9%	60.6%		
Endorse	54.3%	53.1%	54.9%			62.1%	39.4%		
**ACADEMIC BENEFITS**									
**Allow me to earn credit**				3.31	0.069			0.49	0.485
Not endorse	16.2%	21.9%	13.6%			15.2%	18.3%		
Endorse	83.8%	78.1%	86.4%			84.8%	81.7%		
**Allow me to graduate**				3.71	0.054			0.01	0.916
Not endorse	20.5%	27.1%	17.5%			20.7%	20.2%		
Endorse	79.5%	72.9%	82.5%			79.3%	79.8%		
**Number of Anticipated Benefits**	9.01 (±4.06)	8.03 (±3.99)	9.51 (±4.01)	−2.99	0.003	9.28 (±4.14)	8.59 (±3.87)	1.41	0.160

When comparing anticipated general benefits from BPEAC enrollment by sex, a significantly larger proportion of females reported “keep me healthy” (χ^2^ = 18.76, *P* < 0.001) and “improve my health” (χ^2^ = 7.08, *P* = 0.008). For anticipated physical benefits, a significantly larger proportion of females endorsed “help me stay active” (χ^2^ = 10.84, *P* = 0.001), “help control my weight” (χ^2^ = 23.35, *P* < 0.001), and “improve my aerobic fitness level” (χ^2^ = 12.94, *P* < 0.001). For mental reasons, a significantly larger proportion of males reported “help me relax” (χ^2^ = 3.93, *P* = 0.047) as an anticipated benefit from enrolling in BPEAC. Overall, on average, females endorsed significantly more anticipated benefits from enrolling in BPEAC (*t* = −2.99, *P* = 0.003).

When comparing anticipated general benefits from BPEAC enrollment by course type, a significantly larger proportion of those in fitness courses endorsed “keep me healthy” (χ^2^ = 13.69, *P* < 0.001) and “improve my health” (χ^2^ = 23.44, *P* < 0.001). Conversely, a significantly larger proportion of those in sport classes reported “learn new physical activity skills” (χ^2^ = 31.99, *P* < 0.001), “learn about sports” (χ^2^ = 51.08, *P* < 0.001), “provide me with a challenge” (χ^2^ = 13.66, *P* < 0.001), and “allow me to have fun” (χ^2^ = 39.96, *P* < 0.001) as anticipated benefits from enrollment. When comparing anticipated physical benefits from BPEAC enrollment by course type, a significantly larger proportion of those in fitness classes endorsed “help control my weight” (χ^2^ = 25.27, *P* < 0.001) and “improve my aerobic fitness level” (χ^2^ = 9.05, *P* = 0.003). For mental benefits, a significantly larger proportion of those in fitness courses endorsed “improve my self-confidence” (χ^2^ = 4.39, *P* = 0.036) as an anticipated benefit from enrolling in BPEAC. A significantly larger proportion of those in sport classes endorsed all three anticipated social benefits from enrolling in BPEAC.

Kappa statistics indicating correlations between reasons for BPEAC enrollment and anticipated benefits from enrollment are presented in Table 4. For all participants, every Kappa statistic was statistically significant at *P* < 0.001. These Kappa statistics ranged from 0.318 to 0.626. In order of Kappa statistic strength, the highest Kappa statistics were observed for matched items in the mental category (range = 0.495–0.626), which was followed by matched items in the academic category (range = 0.451–0.482). Matched items in the physical and social categories had the weakest Kappa statistics (range = 0.318–0.392). When comparing by sex, stronger Kappa statistics were observed for eight of the 11 matched items relative to their male counterparts. When comparing by course type, the strongest Kappa statistics were more evenly divided between sport and fitness courses (i.e., stronger Kappa statistics for six matched items among students enrolled in fitness courses relative to five matched items among students in sport courses). Those in fitness courses had stronger Kappa statistics for the majority of matched items in the mental and academic categories. Conversely, those in sport courses had stronger Kappa statistics for the majority of matched items in the physical and social categories.

**Table 4:** **Kappa statistics measuring correlation between reasons for course enrollment and anticipated benefits**.

			Sex		Course type	
Reason for enrollment	Anticipated benefits	Total (*n* = 302)	Male (*n* = 96)	Female (*n* = 206)	Sport (*n* = 198)	Fitness (*n* = 104)
**General**
To have fun	Allows me to have fun	0.598***	0.524***	0.629***	0.483***	0.594***
**Physical**
To improve my fitness level (“get in shape”) or (“stay in shape”)	Improves my aerobic fitness level	0.318***	0.223*	0.326***	0.270***	0.358***
To lose weight	Helps control my weight	0.392***	0.504***	0.327***	0.403***	0.280**
To maintain my body weight	Helps control my weight	0.378***	0.378***	0.329***	0.407***	0.269**
**Mental**
To improve my self-confidence	Improves my self-confidence	0.626***	0.510***	0.671***	0.651***	0.592***
To reduce stress/anxiety levels	Reduces my stress	0.573***	0.455***	0.618***	0.538***	0.640***
To relax	Helps me relax	0.495***	0.317**	0.584***	0.479***	0.525***
**Social**
To meet new people	Allows me to meet new people	0.390***	0.319**	0.418***	0.440***	0.222*
**Academic**
To learn a new activity	Learn new physical activity skills	0.482***	0.405***	0.515***	0.501***	0.198*
To earn credit	Allows me to earn credit	0.451***	0.369***	0.498***	0.444***	0.462***
Because it is required to graduate	Allows me to graduate	0.457***	0.567***	0.381***	0.388***	0.597***

*P < 0.05; **P < 0.01; ***P < 0.001.

## Discussion

Findings from this study indicate freshman college students endorsed general, physical, mental, social, and academic reasons for and benefits from enrolling in BPEAC. Findings confirm that more females enroll in fitness classes (26, 34) and suggest that males and females participate in these BPEAC for different reasons and hope to receive different benefits. Consistent with previous research (26, 27, 34), the highest rated reasons why students enrolled in their BPEAC were to earn credit, meet a graduation requirement, exercise regularly, improve their fitness level, and have fun. The highest rated anticipated benefits to students were to stay active, earn credit, graduate, have fun, and keep healthy. There was high concordance between reasons why students enroll in BPEAC and the anticipated benefits from their enrollment.

Compared to males, females reported more reasons for enrolling BPEAC. Females also anticipated receiving more benefits from the course and thought their participation would influence their future physical activity. These findings suggest females may value the ability of physical education courses to increase physical activity levels and maintain fitness or physical benefits. Consistent with previous studies, this study indicates females enrolled in fewer high-school physical education classes and participated in fewer intramural sports than their male counterparts (26). While women in this sample reported being less active than others their same age and gender, males reported being more active than others their same age and gender. Implications from this finding suggest a need to intentionally develop PE programs and courses for women that focus on increasing their overall level of physical activity, but continue to offer high-quality physical activity options that interest men. The variation in reasons between males and females to be engaged in physical activity may justify gender-specific programing to increase female participation.

When examining reasons why students enrolled in BPEAC, females endorsed fitness and exercise as reasons to enroll, while smaller proportions of males endorsed these reasons. When examining anticipated benefits from BPEAC enrollment, a larger proportion of females endorsed graduation and fitness, while a larger proportion of males endorsed more topics including graduation as well as social reasons and enjoyment.

Changes in collegiate graduation requirements bring light to the importance of being aware about factors related to participation in PE courses. The number of 4-year universities requiring students to take PE courses is at its lowest ever. In 1920, 97% of students in 4-year higher education institutions were required to take PE compared to only 39% in 2010 (37, 38). We speculate that the requirement reduction is attributed to increased attention on academics and smaller budgets. Although research documents the connection between academic and life success and physical activity (37), the number of colleges requiring participation continues to decline. At a time when students arrive to college campuses overweight, inactive, and poorly educated about physical health, reducing PE requirements seems counterproductive and harmful to the students’ wellbeing. The lack of graduation requirements for PE emphasizes the need to explore strategies for enticing students to voluntarily participate in BPEAC.

Knowing what motivates students enrollment in BPEAC and the anticipated benefits they expect from them has implications on course development, curricular offerings, and teaching strategies. Motivating students to enroll in physical activity courses and engage in physically active lifestyles is best accomplished by providing choice, interesting activities, and a perception of competence (39). Colleges and universities need to offer a variety of physical activity options, which could include intramural sports and club sports related to the physical education courses to meet the diverse needs and interests of students. Offering students incentives such as extra credit for joining an intramural sports or club team related to their physical education could augment the course while providing additional opportunities to become more active. Courses to develop knowledge and skills for individual life time physical activity, such as cycling, salsa dancing, boot camp training, or self-defense, can have an impact on physical activity levels beyond the collegiate experience. Having a variety of novel choices to develop physical activity competence will provide students with greater motivation to become and continue to remain physically active.

Because both men and women stated that three of their top five reasons for enrolling included to have fun, improve their fitness, and exercise regularly, curricular design needs to focus on methodologies, which allow students to enjoy themselves, and build their knowledge and skills while consistently achieving a moderate level of physical activity. The syllabi should contain course objectives, and daily activities, which reflect students’ needs for the development of physical activity competence and while addressing the desires for enjoyment. Designing courses to meet the student’s perceived goal, to have fun while exercising, functions to build intrinsic motivation to continue participation. Wagner and McNeill (39) suggest that the instructor and planned curriculum have a significant role in ensuring students become intrinsically motivated if lifetime physical activity is to be sustained (39).

A wide range of strategies to increase participation in activity courses exist. This study highlights the desire of females to engage in fitness courses compared to a preference by males for sport courses. Colleges and universities could develop tailored offerings to specifically target males or females. Using gender targeted marketing strategies has the potential to enhance enrollment in those courses, and therefore, increasing the number of physically active college students. An opposite strategy would be to promote enrollment of males in fitness courses using targeted messages related to aspects of competition. Messages to entice female participation in sports courses might emphasis the comradely and social benefits of being on a team while building cardiovascular endurance. Designing and using targeted marketing could be an effective strategy to increase course enrollment.

### Limitations

This study is not without limitations. First, data were self-reported, thus responses may have been biased and responses may have been exaggerated. Actual physical activity levels were not measured, thus the accuracy of involvement and normative perceptions could not be confirmed. The scope of this study was freshman enrolled at one university during one semester, thus findings should not be widely generalized. Future studies should expand the scope to include students from other grade levels, at different schools, and in different parts of the US. This study was cross-sectional, thus there was no follow-up with participants after the class to measure whether or not anticipated benefits were actually received. Future studies could benefit from utilizing a pre-test/post-test study design. While the researchers examined concordance by matching reported reasons and benefits, the wording between items in each category was similar but not exact. This may have introduced some measurement bias.

## Conclusion

This study provides a unique glimpse into the enrollment of basic physical education activity courses (BPEAC) among college freshmen. A larger proportion of females enrolled in fitness courses and a larger proportion of males enrolled in sport courses. A variety of reasons for enrollment were identified as were a variety of anticipated benefits. Reasons and benefits spanned many domains (i.e., general, physical, mental, social, and academic) and were highly correlated. Sex-based differences were observed based on these reasons and anticipated benefits, with females endorsing more fitness-related reasons and benefits compared to male. Findings indicate that these courses can play a role to engage college students in regular physical activity and maintain healthy lifestyles throughout college and beyond. Implications highlight the need for universities to provide quality BPEAC, promote high-quality instruction, and offer a wide variety of physical education courses to meet the diverse needs of students.

## Conflict of Interest Statement

The authors declare that the research was conducted in the absence of any commercial or financial relationships that could be construed as a potential conflict of interest.
